# Structural and immunological basis of cross-reactivity between dengue and Zika infections: Implications in serosurveillance in endemic regions

**DOI:** 10.3389/fmicb.2023.1107496

**Published:** 2023-03-17

**Authors:** Carlos Gaspar-Castillo, Mario H. Rodríguez, Vianney Ortiz-Navarrete, Celia M. Alpuche-Aranda, Jesus Martinez-Barnetche

**Affiliations:** ^1^Center for Infectious Diseases Research, National Institute of Public Health, Cuernavaca, Mexico; ^2^Department of Molecular Biomedicine, Center for Research and Advanced Studies of the National Polytechnic Institute, Mexico City, Mexico

**Keywords:** dengue, Zika, cross-reactivity, antibody, serosurveillance

## Abstract

Dengue and Zika are arthropod-borne viral diseases present in more than 100 countries around the world. In the past decade, Zika emerged causing widespread outbreaks in new regions, where dengue has been endemic-epidemic for a long period. The wide and extensive dissemination of the mosquito vectors, *Aedes aegypti*, and *Ae. albopictus*, favor the co-existence of both infections in the same regions. Together with an important proportion of asymptomatic infections, similar clinical manifestations, and a short time window for acute infection confirmatory tests, it is difficult to differentially estimate both dengue and Zika incidence and prevalence. DENV and ZIKV flavivirus share high structural similarity, inducing a cross-reactive immune response that leads to false positives in serological tests particularly in secondary infections. This results in overestimation of recent Zika outbreaks seroprevalence in dengue endemic regions. In this review, we address the biological basis underlying DENV and ZIKV structural homology; the structural and cellular basis of immunological cross reactivity; and the resulting difficulties in measuring dengue and Zika seroprevalence. Finally, we offer a perspective about the need for more research to improve serological tests performance.

## 1. Introduction

Dengue is the most common arthropod-borne viral disease transmitted by *Aedes aegypti* and *A. albopictus* mosquito vectors. Dengue serocomplex belongs to the genus Flavivirus (*Flaviviridae* family) and is composed of four serotypes DENV 1–4 (DENVs). The endemic circulation of different serotypes, alternating with epidemic cycles, lead to a high disease burden in tropical and subtropical countries. An estimated 390 million annual dengue infections occur globally, of which more than 70% are reported in South-East Asia, 16% in Africa and 14% in Americas (Bhatt et al., 2013). Although 75% of cases are asymptomatic (Bhatt et al., 2013), approximately 2 million progress to severe dengue (Gubler, 2012) that causes life threatening plasma leakage and hemorrhagic complications (Guzman et al., 2016).

Zika virus (ZIKV) is closely related to DENVs (Faye et al., 2014). ZIKV caused extensive outbreaks during the past decade. ZIKV shares the same mosquito vector species as DENVs, so that dengue endemic areas were also susceptible to Zika outbreaks (Duffy et al., 2009; Sanchez, 2016; Aubry et al., 2017). A large outbreak occurred in Yap Islands, Micronesia in 2007, reaching an attack rate of 66% (Aubry et al., 2017). Then, ZIKV spread across the Pacific in 2013–2017, affecting French Polynesia and most of Latin America (Sanchez, 2016; Aubry et al., 2017). Like dengue, up to 80% of Zika cases are asymptomatic, however, its association with rare but important clinical outcomes, including congenital microcephaly, miscarriages and Guillain-Barré Syndrome in adults (Brasil et al., 2016; Cao-Lormeau et al., 2016; Aubry et al., 2017), increases the concern about its transition to an endemic stage.

Most developing countries monitor infectious disease incidence by symptomatic-based surveillance to identify the population at risk, with the intention to focus efforts on control (Murray and Cohen, 2017). In general, this strategy allows the identification of populations more affected by the disease and could guide control efforts (De Vries et al., 2021). However, flaviviral infection incidence is considerably underreported due to the high proportion of asymptomatic and mild disease cases, and similar clinical manifestations confound the etiologic diagnosis (Ioos et al., 2014). Pathogen detection by molecular testing (qPCR) or antigen detection (i.e., NS1) have a good performance only during a short-time window (Hunsperger et al., 2016; Judice et al., 2018) and the increasing number of private low-cost healthcare facilities with a questionable level of participation in the disease surveillance system (Berntsen and Syverud, 2009; Ahmadi et al., 2013). Thus, underreporting represents a missed opportunity to stablish a reliable denominator to monitor epidemic dynamics.

Serological surveys provide better estimations of the exposure to pathogens. For many infectious diseases, pathogen-specific IgG population-based seroprevalence estimation is a good epidemiological tool that provides a denominator to calculate pathogen exposure as indirect measure of incidence, independently of clinical manifestations and access to the health system (Cutts and Hanson, 2016). The capability of IgG to persist for a long time in bloodstream (Guzmán and Kourí, 2004), makes it a reliable biomarker that provides useful indicators for retrospective monitoring of the epidemic magnitude. Moreover, seroprevalence estimation, particularity in the context of dengue, could contribute to guide prioritization of vaccination based on previous exposure (WHO, Immunization, Vaccines, and Biologicals, 2017; Aguiar and Stollenwerk, 2018; WHO, 2019).

However, DENVs and ZIKV share a high degree of protein structural similarity, including the NS1 and E proteins, which are commonly used as antigens in indirect serological tests aimed to measure virus-specific IgG. Moreover, the induced B cell repertoire contains highly cross-reactive antibodies (crAbs), which does not correlate with their neutralizing capacity and breadth, affecting the diagnostic performance of serological tests and the consequent inaccurate seroprevalence estimation. Most of the commercial tests designed after the Zika outbreaks in the Americas were validated in volunteers or travelers who lived in non-endemic areas. However, they have poor performance for dengue and Zika detection in dengue endemic areas, although in dengue non-endemic regions the specificity is above 90% (Table 1).

**Table 1:** Diagnostic sensibility and specificity of dengue and Zika serological tests obtained in endemic and non-endemic regions.

Test	Flaviviral primo infeccion				Secondary flavivirus infeccion			
	Population	Sensitivity [IC95%]	Specificity [IC95%]	References	Population	Sensitivity [IC95%]	Specificity [IC95%]	References
Based on total antibodies								
DENV-NS1 IgG ELISA	Thai children	56.4% [52–61]	95.3% [91, 98]	Blacksell et al. (2012)	Mexican adults	100% [100, 100]	85% [65.6–100]	Zaidi et al. (2019)
ZIKV-NS1 IgG ELISA	Canadian adults	23.7% [14.1–33.2]	95.2% [91.8, 98.7]	L’Huillier et al. (2017)	ND	Travelers who living in non-endemic areas	54% [26.9, 81]	Van Meer Maurits et al. (2017)
ZIKV-NS1 IgG BOB	Nicaraguan, Brazilian, England and Italian adults	91.8% [87.5, 96]	88.10% [83.9, 93.9]	Balmaseda et al. (2017)	Nicaraguan, Brazilian, England and Italian adults	100% [100, 100]	80.4% [71.1, 87.8]	Balmaseda et al. (2017)
ZIKV-EDIII IgG ELISA	French Antilles	92% [84.5, 99.5]	90% [84.0, 95.8]	Denis et al. (2019)	French Antilles	ND	90% [84.1–95.9]	Denis et al. (2019)
quadruple mutant of DENV-FL IgG ELISA	European travelers returning from endemic areas	100% [63.4, 100]	97.1% [85.1, 99.9]	Rockstroh et al. (2017)	ND	ND	ND	ND
quadruple mutant of ZIKV-FL IgG ELISA	European travelers returning from endemic areas	100% [81.5, 100]	97.9% [92.6, 99.7]	Rockstroh et al. (2017)	ND	ND	ND	ND
ZIKV-NS1 IgG MIA	Taiwanese, Nicaraguan, Hawaii	100% [100, 100]	87.9% [82.9, 90.4]	Tyson et al. (2019)	Taiwanese, Nicaraguan, Hawaii	100% [100, 100]	54.6% [40.8, 70.2]	Tyson et al. (2019)
DENV-NS1 IgG MIA	Taiwanese, Nicaraguan, Hawaii	94.3% [89.4, 96.8]	97.2% [94.1, 98.8]	Tyson et al. (2019)	Taiwanese, Nicaraguan, Hawaii	94.3% [89.4, 96.8]	94.7% [87.6, 100]	Tyson et al. (2019)
DENV IIFT	Dengue patients	87.1% [70.2, 96.4]	91.7% [86.5, 95.4]	Arai et al. (2020)	ND	ND	ND	ND
DENV RDT	Dengue patients	96.8% [94.2, 98.4]	100% [98.8, 100]	Lee et al. (2015)	Mexican patients with febrile illness	90.1% [85.3, 94.8]	92.5% [88.8, 96.1]	Sánchez-Vargas et al. (2014)
Based on Neutralizing antibodies								
ZIKV PRNT_50_	Latin American patients	96.8% [83.3, 99.9]	95% [75.1, 99.9]	Loyola et al. (2021)	Latin American patients	96.7% [82.8, 99.9]	93.3% [68.1, 99.8]	Loyola et al. (2021)
DENV PRNT_50_ (DENV-1 to −4)	French Polynesian patients	100% [47.82, 100]	100% [47.82, 100]	Lanciotti et al. (2008)	French Polynesian patients	100% [59.03, 100]	57.1% [20.4, 93.7]	Lanciotti et al. (2008)
DENV-1, −2 MNT	Vaccinated Volunteers	100% [96.19, 100]	93.8% [85.4, 100]	Putnak et al. (2008)	ND	ND	ND	ND
DENV-2 MNT	Vaccinated Volunteers	100% [96.19, 100]	96.8% [90.7, 100]	Putnak et al. (2008)	ND	ND	ND	ND
DENV-3, 4 MNT	Vaccinated Volunteers	100% [96.19, 100]	100% [96.19, 100]	Putnak et al. (2008)	ND	ND	ND	ND
ZIKV MNT50	French overseas patients	85% [72.9, 92.4]	98.7% [92.4, 99.9]	Nurtop et al. (2018)	ND	ND	ND	ND
DENV RVNT	Travelers who living in non-endemic areas	100% [38.3–100]	96.6% [90.0, 99.3]	Shan et al. (2017)	ND	ND	ND	ND
ZIKV RVNT	Travelers who living in non-endemic areas	100% [74.9, 100]	94.3% [88.4, 97.4]	Shan et al. (2017)	ND	ND	ND	ND

ND, Data not available.

This integrative review aims to provide a broad overview of the structural and immunological basis of the cross-reactive antibody response to dengue and Zika infections, and their implications in epidemiological serosurveillance, as well as insights into the design of novel, more specific serological tests.

## 2. Dengue and Zika phylogeny

Evidence points to DENVs originating from sylvatic strains infecting non-human primates and many forest-dwelling *Aedes* vectors different from *Ae. aegypti* and *Ae. albopictus* (Rudnick, 1965; Robin et al., 1980). Phylogenetic analyses of E protein sequences of historical sylvatic and endemic isolates of DENV suggests that the ancestor of all DENV could have originated in West Africa (Rudnick, 1965; Robin et al., 1980), approximately 1,000 years ago (Rambaut, 2000; Twiddy et al., 2003). Endemic DENV-2 genotypes diverged from West African sylvatic strains about 400–600 years ago, and then sylvatic and epidemic DENV-1 and -4 strains diverged around 100–200 years ago (Twiddy et al., 2003; Weaver and Vasilakis, 2009). Slave trade from Africa to other continents and more recently, human mobility, urbanization and trade routes have contributed to global DENV spread and diversification (Smith, 1956).

ZIKV was first isolated in Uganda in 1947 from a rhesus monkey and its origin is still unclear, mainly due to limited sampling. Like DENV, ZIKV could have originated in a widely enzootic cycle among multiple animal species in Africa (Gutiérrez-Bugallo et al., 2019). Phylogenetic analyses of E and NS5 sequences showed that the common ancestor of ZIKV diverged from Spondweni virus before 1800s (Pettersson et al., 2016), and the common ancestor of all ZIKV lineages could have originated at the beginning of 1900s, forming the Ugandan lineage. This lineage probably spread in West African countries around 1935 (West African lineage), and in South East Asia around 1945. ZIKV reached Micronesia around 1960 (Asian lineage) (Faye et al., 2014). The pathway of ZIKV dissemination from Africa to other continents is unclear. The recent emergence of Zika in Brazil was associated with the massive movement and gathering of people motivated by the FIFA Soccer World Cup or Canoe Sprint World Championships in 2014 (Faria et al., 2016), supporting human movement and trade routes as major determinants for the rapid spread of Zika.

**Figure 1:**
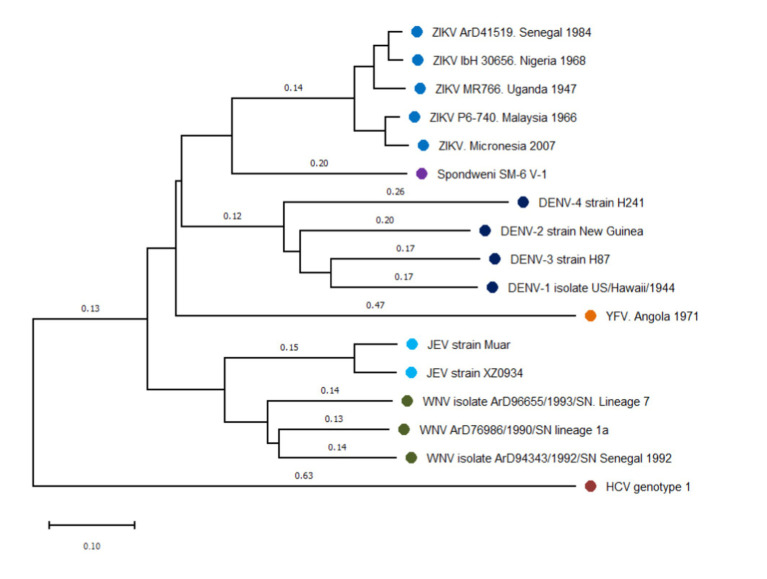
Phylogenetic N-J tree of flaviviruses related to dengue and Zika virus. The phylogeny was represented using reference sequences of different flaviviruses, showing the close relationship among dengue (DENV) and Zika virus (ZIKV). Four dengue serotypes form the dengue serocomplex, that is presented in this tree by DENV-1 (EU848545), DENV-2 (AF038403), DENV-3 (M93130), and DENV-4 (AY947539). ZIKV and Spondwenii virus constitute a Spondwenii serocomplex, that is represented by the first ZIKV isolated from rhesus monkey (NC_012532), two strains of ZIKV West African lineage: ArD41519 (HQ234501) and ibH30656 (HQ234500) and two of Asian lineage P6-740 (HQ234499) and isolated strains from Micronesia 2007 (U545988). Spondwenii virus was represented for SM-6 V-1 strain (DQ859064). Japanese Encephalitis serocomplex was represented for Japanese Encephalitis virus (JEV) Muar (HM596272) and XZ0934 strains (JF915894), and West Nile Virus (WNV) lineage 7 (KY703855), lineage 1a (KY703854), and strains from Senegal, 1992 (KY703856). Yellow Fever virus (YFV) forms a distinct group, represented by AY968064. Hepatitis C virus (HCV) strain (NC_004102) was used to outroot the tree. Distance represents base substitutions per site.

There is little evidence to estimate a precise divergence time between DENVs and ZIKV, but it is plausible that divergence occurred before 1,000 years ago, when the common ancestor of DENVs originated (Figure 1). It is also proposed that ZIKV could have diverged from DENVs, based on the close relationship between ZIKV and DENV-4 (Ratfi and Nizam, 2016), implicating that ZIKV origin is more recent. Nevertheless, both theories point to a relatively short divergence time, which explains the high structural similarity between both viruses (Barba-Spaeth et al., 2016).

## 3. DENV and ZIKV structure

**Figure 2:**
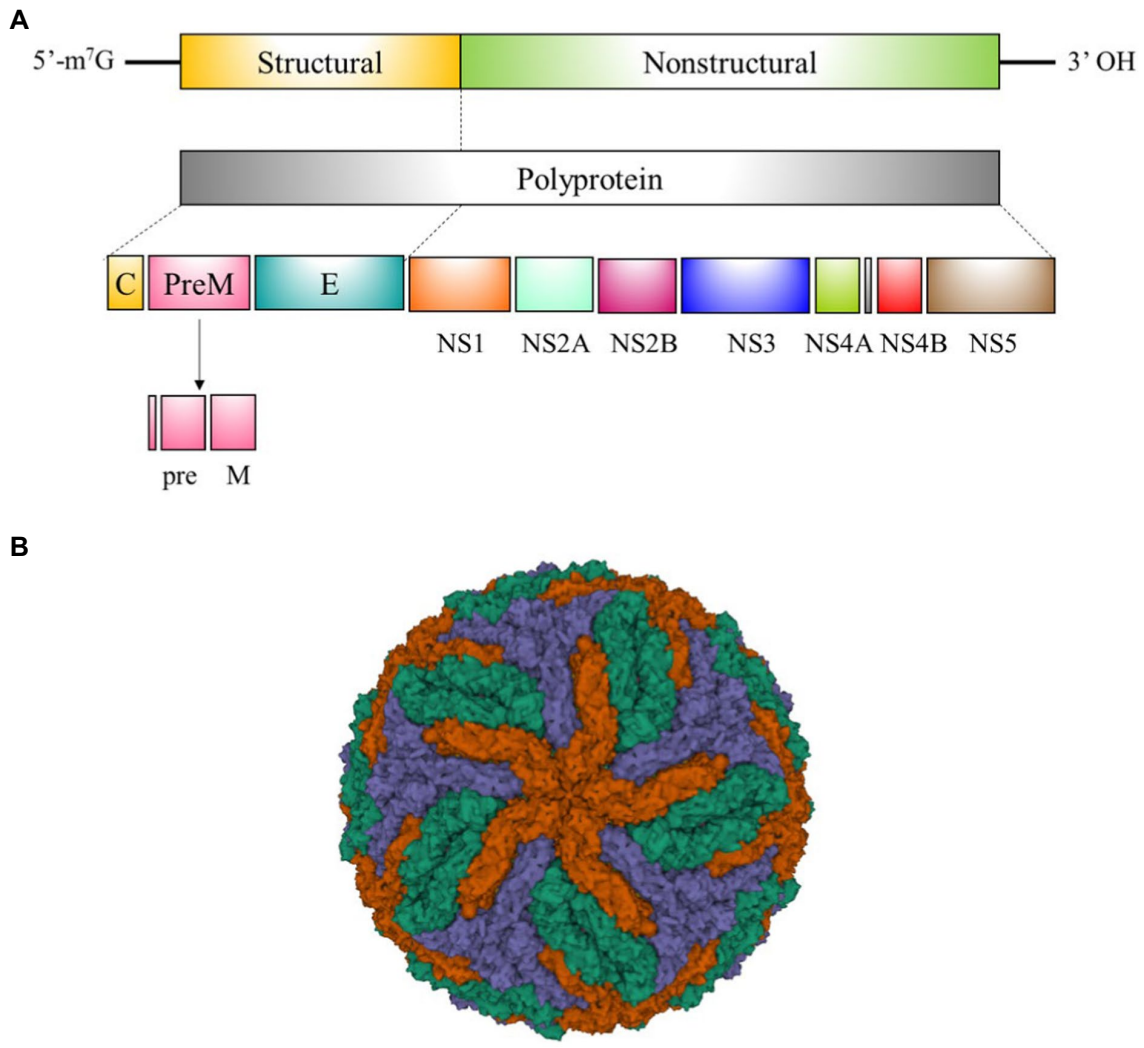
Flavivirus genome organization and structure. **(A)** Schematic representation of flavivirus genome organization and processing of the polypeptide into mature viral proteins. **(B)** Cryogenic electron microscopy structure of the dengue virus (DENV). E:M heterodimers of the same color are equivalent by icosahedral symmetry. Heterodimers of different colors are quasi-equivalent, with red E:M dimers falling on the icosahedral 5-fold axes, blue on the 3-fold, and green on the 2-fold (PDB 3 J27) (Zhang et al., 2013).

Flaviviruses are enveloped single-stranded positive-sense RNA viruses that share the same basic genomic organization and molecular structure (Lindenbach et al., 2011). RNA genome of flaviviruses encode a single polyprotein, which is cleaved into seven non-structural (NS) proteins (NS1, NS2A, NS2B, NS3, NS4A, NS4B, and NS5) implicated in controlling critical steps of the viral life cycle; and three structural proteins (the capsid [C], precursor membrane [prM] and envelope protein [E]) constituents of the viral particle (Figures 2A,B; Lindenbach et al., 2011). This review is focused on NS1 and E, both highly immunodominant antigens used as targets for antibody detection in the majority of serological tests.

### 3.1. NS1 protein

**Figure 3:**
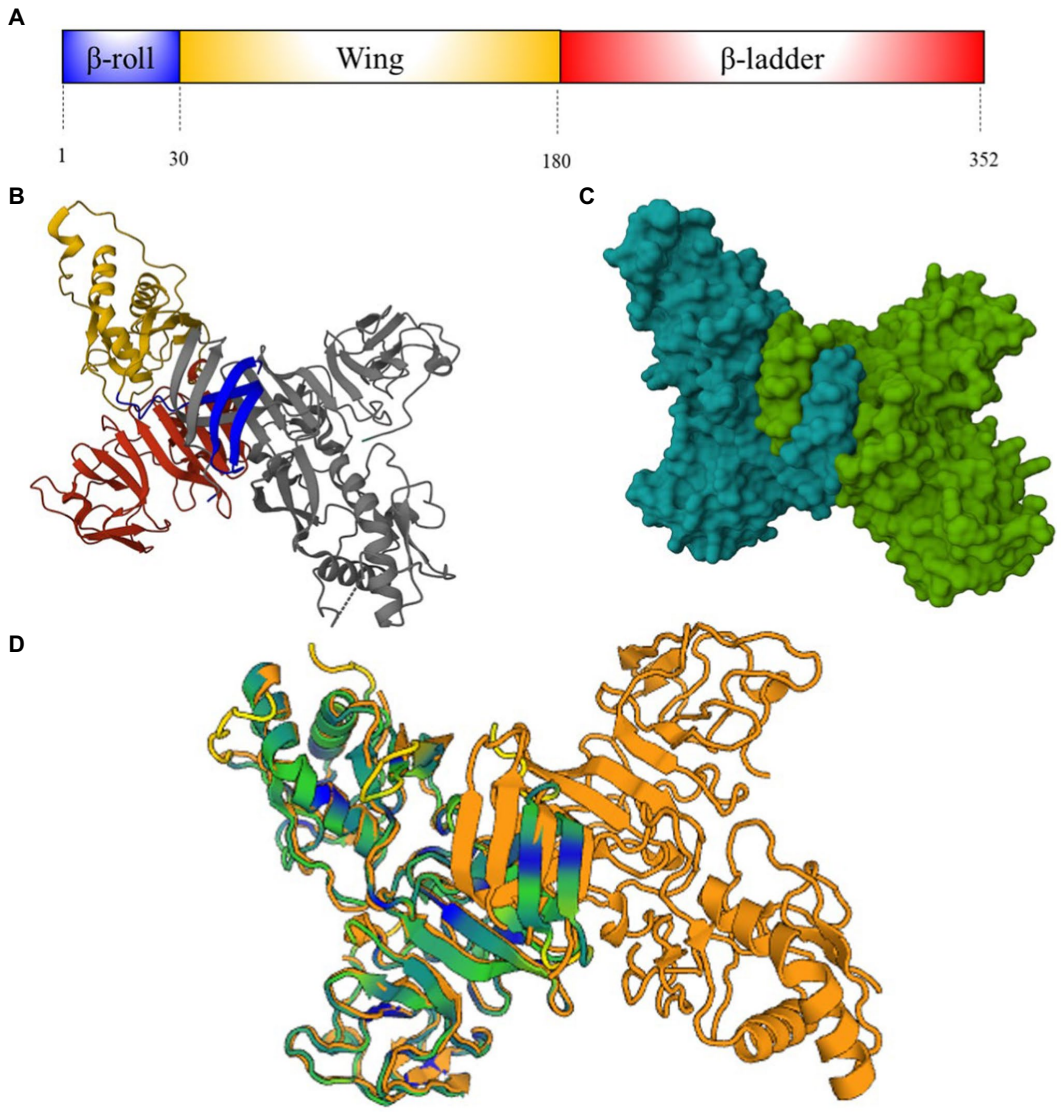
NS1 protein structure. **(A)** Schematic representation of ZIKV NS1-protein showing β-roll (blue), wing (yellow) and β-ladder domain (red). Numbers below represent the residue positions **(B)** Ribbon molecular structure of ZIKV NS1 cross-shaped dimer (PDB: 5K6K) (Brown et al., 2016). Domains on one monomer are represented as **(A)**, another domain is showed in gray. **(C)** Molecular structure of ZIKV NS1 cross-shaped dimer (PDB 5K6K). **(D)** DENV (4O6B) and ZIKV (5K6K) NS1 superposition [Dali server: (Holm, 2022)]. ZIKV-NS1 sequence A (green) is super posited on DENV-2-NS1 sequence A and B (orange) (Akey and Smith, 2014). Cyan and blue regions indicate conserved motifs and conserved amino acid sequences.

NS1 is highly conserved among flaviviruses. It is constituted by around 352 amino acids and has a molecular weight of between 46 and 55 kDa. The NS1 of most flaviviruses has N-linked glycosylation sites at asparagine 130 and 207 and cysteine residues which form disulfide bonds at the carboxy terminal region. The NS1 contains three distinct domains: A N-terminal β-roll, a wing domain, and a C-terminal β-ladder, that have approximately a amino acid sequence of 30, 150, and 170 residues, respectively (Figure 3A; Akey et al., 2014).

NS1 is synthesized in the endoplasmic reticulum (ER) as a monomer is anchored in the plasma membrane as a dimer. The C-terminal disulfide bonds of this protein are crucial for dimerization. NS1 dimers are cross-shaped attached by the intertwined β-roll domains, the β-ladders interact with each other, and the wing domains are end to end (Figures 3B,C). The quaternary structure of NS1 on the cellular membrane has two faces: The hydrophilic “outer” face that contains glycosylation sites and the hydrophobic “inner” face that belongs to the β-roll domain and an adjacent “greasy finger” loop. The NS1 is secreted as hexamer, in a barrel-like structure, attached by the hydrophobic inner face (Youn et al., 2012).

In the particular case of DENVs and ZIKV, alignment of their NS1 protein sequences reveals 51–53% similarity (Wen and Shresta, 2019). Crystal structure of ZIKV-NS1 showed differences on the wing domain flexible loop (residues 108–129) that contain three highly conserved tryptophan residues (residues 123–124) on the inner hydrophobic face. Together with other tryptophan residues on the wing domain and greasy fingers loop form a hydrophobic protrusion, which is an additional site for cellular membrane interaction (Brown et al., 2016). Negative charged regions displayed on the wing and central β-ladder domains of the outer hydrophilic face of ZIKV-NS1 are absent in DENV-2 and West Nile Virus (WNV) NS1. These differences do not modify the protein folding and the hexameric configuration of NS1 among DENVs and ZIKV (Figure 3D), although they could represent differential sites for antibody recognition and vaccines designing (Akey et al., 2014).

### 3.2. E protein

**Figure 4:**
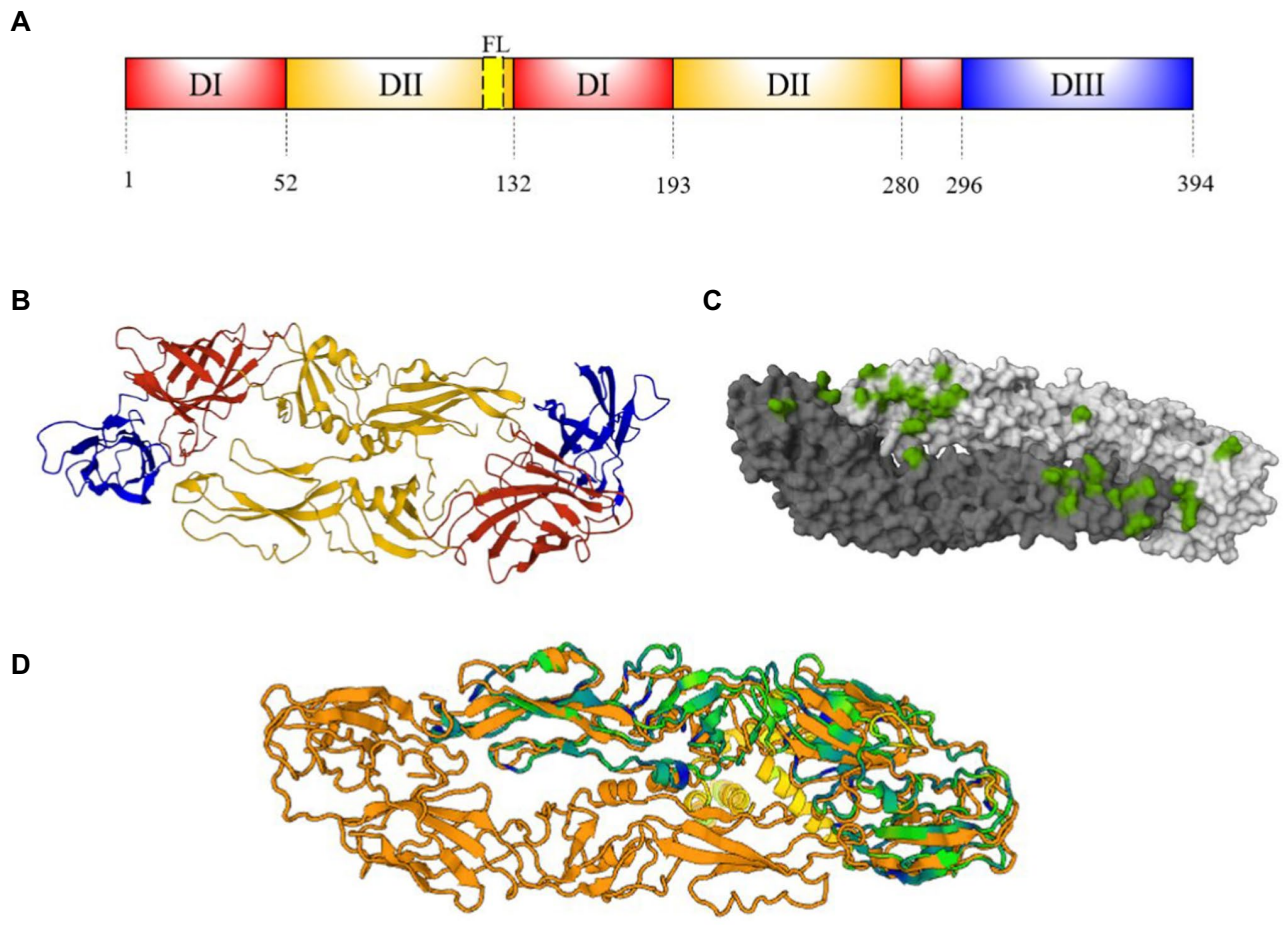
E protein structure. **(A)** Schematic representation of E-protein ectodomain showing EDI (red), EDII (orange), FL (yellow) and EDIII (blue). Numbers below represent the residue positions. **(B)** Ribbon structure of DENV E antiparallel dimer (PDB: 1UZG) (Modis et al., 2005). Domains on dimer are represented as **(A,C)** Footprint of EDE2 Fab C8 mAb on a DENV-2 antiparallel dimer (4UTC) representation shown in green (Barba-Spaeth et al., 2016). **(D)** DENV (1UZG) and ZIKV (5IRE) E dimer superposition [Dali server: (Holm, 2022)]. ZIKV-NS1 sequence A (green) (Sirohi et al., 2016) is super posited on DENV-3-E sequence A and B (orange). Cyan and blue regions indicate conserved motifs and conserved amino acid sequences, yellow motifs indicate DENV-E intracellular regions.

The E protein is highly conserved among flaviviruses. The monomer consists of approximately 500 residues and has a molecular weight of 50 kDa (Dai et al., 2016). DENV and ZIKV E protein contains one or two glycosylated asparagine residues (Asn 67 and/or Asn153-154) and α-helices at C- terminus (Dai et al., 2016). For both viruses, E is formed by three different domains (EDI-EDIII). The EDI is a central ß-barrel domain with 130 residues in three segments (residues 1–51, 132–192, and 280–295) and contains a glycan loop (GL, residues 147–161). The EDII has a finger-like conformation formed by two segments (residues 52–131 and 193–279), that include a highly hydrophobic fusion loop (FL, residues 98–109); and EDIII has a immunoglobulin-like fold (residues 296–403; Figure 4A; Dai et al., 2016).

The E proteins are assembled in 60 trimeric protrusions anchored to preM in ER, forming immature virions with spiky surfaces (Lindenbach et al., 2011). During the virus maturation, low pH in trans-Golgi networks induces a reorganization of E-preM heterodimers, producing 90 antiparallel E homodimers (Figure 2B), connected through EDII and EIII (Figures 4B,C). This organization produces the exposure and cleavage of pre-M and herringbone pattern formed by E homodimers in the mature virion (Lindenbach et al., 2011).

The EDI, EDII, and EDIII of ZIKV and DENV share 35, 51, and 29% amino acid similarity, respectively (Dai et al., 2016). Structural studies on flavivirus E protein reported a N67 glycosylation motif on EDI present only in DENV. The N164 glycosylation motif is only present at ZIKV-GL. The latter is larger than DENV, due to the presence of four additional amino acids (Sirohi et al., 2016). The highly similarity into EDII is brought by FL, which is highly conserved among flaviviruses (Stettler et al., 2016). On the other hand, EDIII of ZIKV and DENVs differs in 52 residues, including three additional amino acids. It also includes surface-exposed, internally facing and cryptic residues not displayed in DENVs (Conlan et al., 2019). The E protein quaternary structure is identical in both mature virion species (Figure 4D; Sirohi et al., 2016). Thus, the reduced similarity between EDIII of DENV and ZIKV make it an attractive antigen candidate to improve specificity of serological assays aimed at discriminating seroprevalence between the two infections.

## 4. Humoral immune response against viruses

### 4.1. Structural basis of antibody mediated antigen recognition, specificity, and cross-reactivity

Antibodies are soluble forms of the membrane anchored B cell antigen receptor (BCR). Each naïve B cell bears a unique BCR and ligand (antigen) - binding capacity, which can be clonally selected by the cognate antigen, resulting in antibody secretion by alternative splicing of the BCR transcript. Antigen specificity and cross-reactivity depend on antigen binding site (paratope) structure. The paratope is composed by three hypervariable loops called Complementarity Determining Regions (CDR-H1, -H2, and -H3) of the Heavy and 3 CDR’s of the Light chain, at the Variable region of both chains. CDR1 and 2 have limited variability because they are encoded in germline V segments, while the CDR3 structure is highly variable because it is determined by V(D)J recombination (Davis and Bjorkman, 1988; Schroeder and Cavacini, 2010).

All known CDR1, 2 and CDR-L3 adopt a limited number of standard canonical structures (Chothia and Lesk, 1987; Lara-Ochoa et al., 1996). Only the CDR-H3 loop, is highly diverse and does not adopt a canonical structure. Current evidence suggests that all CDR’s, except CDR-H3, are rather cross-reactive structures that contribute to stabilize the antigen-paratope interaction, and the major determinant for the affinity is the CDR-H3 (Davis, 2004). Under this model, an epitope will be cross-reactive if critical residues that bind to the CDR-H3 are conserved in a variant epitope, despite little or non-conservation of contact residues of the remaining CDR’s. Conversely, variant-specific epitope recognition will occur if the variant epitope has mutated in critical residues that bind to the CDR-H3, despite conservation of the remaining CDR contacts.

A common denominator in some viral infections such as HIV, influenza and flaviviruses is the rapid antigenic variation of the causative agent and the consequent escape from neutralizing antibodies. Yet, in such cases, a small fraction of the responding B cells have strong biases in the use of particular VH segments (Gorny et al., 2009; Wrammert et al., 2011; Wu et al., 2011; Lingwood et al., 2012; Cortina-Ceballos et al., 2015; Dunand and Wilson, 2015; Godoy-Lozano et al., 2016; Nielsen et al., 2020; Robbiani et al., 2020; Voss et al., 2021). These “stereotyped” responses are produced in genetically unrelated individuals and are directed toward highly conserved viral epitopes (structural convergence), hence these are cross-reactive and in some cases, broadly protective. This type of epitope recognition differs with the model proposed by Davis, because the contribution of the germline encoded CDR1 and 2 to overall affinity is higher, explaining the biased V-gene segment use. Thus, germline conservation of certain CDR structures may be an additional source of broad, “innate” cross-reactivity against several viral capsid epitopes.

### 4.2. The role of somatic hypermutation in antibody cross-reactivity

Somatic hypermutation (SHM) is an antigen and T-cell dependent (TD) process that promotes genetic diversification of the V-region followed by affinity-dependent selection of B cells. SHM tends to prevent cross-reactivity by fine-tuning antigen-paratope interaction (Davis, 2004). Affinity matured antibody responses toward TD antigens have important implications for population immunity because they are associated with germinal center (GC) response as the major site of antibody affinity maturation, class switch and the generation of long lived plasma cells (LLPC) and memory B (mB) cells (Goodnow et al., 2010).

However, repeated antigen stimulation and the consequent extensive SHM, in the GC pathway may also contribute to cross-reactivity and polyreactivity as has been demonstrated in HIV (Gorny et al., 2009; Wu et al., 2011) and possibly is implicated in dengue progressively cross-reactive and cross-protective response after repeated infections with different serotypes (Zompi et al., 2012; Priyamvada et al., 2016; Patel et al., 2017; Durham et al., 2019).

Geometrically arrayed protein multimers included in viral capsids (Figure 2B), are capable of strong BCR crosslinking in a T-independent (TI) fashion (Zinkernagel, 1996). Thus, in these cases, in the absence of SHM and GC formation, responses against TI antigens are more cross-reactive than TD responses because increased avidity of the interaction.

In recent years, it has become clear that the extrafollicular response (GC-independent) is an important source of non-classical mB cells and plasmablasts secreting cross-reactive unmutated IgG. Thus, EF contributes to cross-reactivity mainly through stereotyped use of certain V-gene segments. Although viral infections induce EF and GC responses, for some acute viral infections that progress to severe systemic inflammation such as severe dengue and severe COVID-19, suppression of GC reaction occurs (Aye et al., 2014; Kaneko et al., 2020), thereby promoting cross-reactivity.

## 5. Cross-reactive antibodies in dengue and Zika

Antibody responses to flavivirus are guided to both structural and non-structural proteins, including NS1, NS3, NS5, pre-M, C, and E proteins (Falconi-Agapito et al., 2021; Khare and Kuhn, 2022). The NS1 and E proteins are targets of neutralizing, non-neutralizing protective and enhancing antibodies, so most research related to the antibody response, including cross reactivity, is focused on them. Only NS1 and E are used as antigenic targets in the available serological tests (Balmaseda et al., 2017; L’Huillier et al., 2017; Safronetz et al., 2017; Lee et al., 2018; Denis et al., 2019; Tyson et al., 2019).

High-throughput B cell repertoire sequencing in acute and post-convalescent dengue infections show a marked stereotyped B cell response with important dominance of VH1 and 3 family segments. The precise antigen causing such stereotyped responses is still unknown (Parameswaran et al., 2013; Godoy-Lozano et al., 2016). More recently, using scRNA-seq coupled to BCR sequencing, a disproportionate use of IGHV1-69 against DENV non-FL epitopes in EF derived plasmablasts was described in primary and secondary dengue infection (Rouers et al., 2021). Similarly, mAbs against ZIKV using unmutated V-gene segments have been described (Magnani et al., 2017; Gao et al., 2019), indicating that the immune response to ZIKV can be stereotyped and may contribute to cross-reactivity.

### 5.1. Antibodies to E domains

In flaviviruses, exposed surfaces are commonly more variable and immunodominant than conserved cryptic domains, thus strong neutralizing Abs (nAbs) are usually type-specific (i.e., non-cross reactive), whereas weak nAbs recognize cryptic epitopes that are highly cross-reactive (Campos et al., 2018). A low proportion of the potent and highly specific nAbs are elicited to EDIII of DENV and ZIKV (Wahala et al., 2009; Gallichotte et al., 2015). Stereotyped responses of anti-ZIKV EDIII antibodies using IGHV3-23/IGKV1-5 are cross-neutralizing for ZIKV and DENV-1, whereas those using different IGHV segments only neutralize ZIKV (Robbiani et al., 2017).

In contrast, a high proportion of poorly neutralizing Abs are directed to the highly conserved EDI/EDII interphase and the FL (EDII), and are highly cross reactive (Priyamvada et al., 2017; Montoya et al., 2018; Premkumar et al., 2018). In fact, cross reactivity and poor neutralizing capacity of anti-FL antibodies are associated with antibody-dependent enhancement (ADE) (Rey et al., 2018), which is associated with heterologous serotype infections (Montoya et al., 2018).

The dimerization on the E monomers creates conformational epitopes along the dimer interphase called EDE-1 and EDE-2 (Figure 4C; Dejnirattisai et al., 2014; Rouvinski et al., 2015). These epitopes are highly conserved within the EDI/DIII–EDII interphase. Antibodies toward conformational epitopes are cross-neutralizing to both, DENV and ZIKV (Dejnirattisai et al., 2014; Rouvinski et al., 2015). Anti-EDE-1 antibody binding is not affected by protein E N153 glycosylation, whereas binding to EDE-2 is dependent on glycosylation. Both types of antibodies potently neutralize DENV, and anti-EDE-1 also strongly neutralizes ZIKV (Dejnirattisai et al., 2016).

Studies in influenza A virus neutralization indicate that the human B cell repertoire generates antibodies capable of broad neutralization targeting cryptic, highly conserved, conformational epitopes, suggesting that such lymphocytes have a selective disadvantage compared to type-specific B cell clones targeting type-specific epitopes (Wrammert et al., 2011; Guthmiller et al., 2020). Studies in hyperendemic dengue regions indicate that the generation of broadly neutralizing responses depend on sequential reinfection by different serotypes (Duffy et al., 2009; Guerbois et al., 2016; Aubry et al., 2017). After a primary infection, most neutralizing B cells are type-specific, and cross-neutralizing mB cells are rare. Subsequent heterologous reinfections induce the recruitment of such mB cells to GC’s, reshaping specificity by SHM. After two or more heterologous reinfections initial subdominant cross neutralizing mB cells start predominating. Thus, as in HIV infection under highly active antiretroviral therapy (HAART) (Gorny et al., 2009; Wu et al., 2011) in recurrent dengue infections GC’s are central for a slowly evolving clonal selection process that results in a broadly neutralizing response (Zompi et al., 2012; Priyamvada et al., 2016; Patel et al., 2017; Durham et al., 2019).

Recent studies have shown that there is a highly cross-reactive antibody and mB cell responses against E in convalescent ZIKV infection in people with previous DENV infection, but that cross-reactivity wanes rather quickly, after which the response becomes ZIKV-specific (Collins et al., 2017; Andrade et al., 2019). These findings suggest that despite the structural similarity, ZIKV should be considered apart from the DENV serocomplex (Collins et al., 2017).

### 5.2. Antibodies to NS1

Anti-NS1 antibodies can confer Fcγ-mediated protection and inhibit severe flavivirus infections through Fcγ-receptor mediated cytotoxicity (Schlesinger et al., 1993; Krishna et al., 2009), complement activation, and other undetermined Fc-independent mechanisms (Chung et al., 2006, 2007). However, anti-NS1 antibodies also participate in severe dengue pathogenesis by promoting bleeding diathesis through inhibition of thrombin activity and enhancement of fibrinolysis (Chuang et al., 2014, 2016).

Most antibodies directed to the wing domain of anti-NS1 protein are cross-reactive, as seen with mouse monoclonal antibodies (mAb), which cross-react with DENVs, JEV and Kunjin virus (Falconar and Young, 1991). Other crAbs directed against the NS1 β-ladder, cross-react with ZIKV and DENV-2 (Falconar and Young, 1991).

Current evidence about pan-anti-flavivirus antibodies has contributed to our knowledge about the antigen-binding sites of antibodies elicited by NS1. The 2B7 human mAb binds to overlapped epitopes at the β-ladder region on the “inner” face and at the wing domain of the protein, blocking NS1 binding to endothelial cells (Biering et al., 2021). CDR-H3 recognition of highly conserved residues, particularly R299 and T301 on NS1 represents a highly cross-reactive response among all dengue serotypes, ZIKV and WNV, despite the rest of CDRs-binding sites are non-conserved among flavivirus (Biering et al., 2021). In contrast, 22NS1, a mouse mAb against the WNV NS1-β-ladder, is non-protective against other flavivirus. The basis of such specificity is the lack of conservation of critical contact sites of the CDR-H3, despite some non-CDR-H3 contact sites being conserved among other flaviviruses (Edeling et al., 2014).

## 6. Implications of cross-reactivity in serological tests for seroprevalence estimation

Accuracy of different diagnostic tests depend on the infection stage. During the viremia, i.e., the first 5–7 days posterior to the infection onset. Specific RNA can be detected by qPCR in blood, serum and plasma, with specificities above to 95%. NS1 can also be detected, although its high similarity makes it difficult to discern among flaviviral infections (Brown et al., 2016; Wen and Shresta, 2019). Anti-flavivirus IgM titres in acute sera are measured to serologically confirm infection, however its short half-life (<120 days posterior onset infection) limits their applicability to a biomarker of active infections and extended cross-reactivity is also present in these serological tests. In contrast, IgGs achieve measurable levels at around seven days posterior the infection onset and persist for more than 3 years in bloodstream (Nascimento et al., 2018). Thus, for clinical diagnosis, IgGs detection is useless the during acute phase, but their high concentration and long half-life make them the most commonly used antibodies in seroprevalence studies (Siegrist, 2018).

In addition, the determination of IgG as a marker of DENV pre-exposure has been considered relevant in studies of the dengue vaccine Dengvaxia^®^ since the evidence of an excess risk of severe dengue in seronegative vaccine recipients compared to seropositive non-vaccinated individuals (Aguiar and Stollenwerk, 2018; WHO, 2019).

The implementation of tests in epidemiological surveillance requires a rigorous validation to determine intrinsic capabilities, i.e., the diagnostic sensibility (DSe) and diagnostic specificity (DSp) (Trevethan, 2017). High DSp increases the likelihood of a true-negative result (Trevethan, 2017), which is ideal when the goal is to discriminate between past infections, for example to determinate seroprevalences in scenarios of dengue endemic regions with recent Zika outbreaks.

A growing number of Enzyme-Linked Immunosorbent Assays (ELISA) based on anti-NS1 IgG detection that are available in the market (L’Huillier et al., 2017; Safronetz et al., 2017), are summarized in Table 1. However, NS1 and E preparations used in commercial ELISA, may contain highly conserved epitopes that may detect crAbs binding, affecting the analytical specificity.

A blockade-of-binding (BOB)-ELISA, which is a competitive ligand-binding assay, produces better results than conventional ELISAs. Using a mAb to ZIKV-NS1 (ZKA3), showed an overall DSp of 80.4% in a panel of secondary dengue infections and 95.9% in dengue patients and healthy volunteers (Balmaseda et al., 2017). Although the ZKA3 epitope is not fully revealed, defined as S2 (Stettler et al., 2016), it is likely that it binds to a non-conserved NS1 region among flavivirus, highlighting the applicability of type specific Abs to improve prevalence estimation. However, there is still much room for specificity improvement, which will require testing novel, more specific epitopes.

Other techniques are being tested to improve performance. The magnetic immunoassay (MIA) utilizes microspheres coated with NS1 (Beck et al., 2015). In primary infections, anti-NS1 IgG MIA showed a DSp of 86–87.9% (Wong et al., 2017) and 97.2% (Tyson et al., 2019) for ZIKV and DENVs, respectively. However, in secondary dengue infections the DSp of ZIKV-NS1 MIA dropped to 54.6%, while it performed better (>90%) for DENVs (Tyson et al., 2019). It is possible that NS1 antigen coupling facilitates the exposure of antigenic hydrophilic epitopes. The NS1 array around beads can also favor better antibody binding than that obtained in solid-phase of conventional ELISA. The analytical specificity is improved for paired antigen of DENVs and ZIKV running out in the same well, and the limit of detection is improved with more resolution using fluorescence intensity than optical densities of ELISA. High-throughput and less clinical volume samples makes MIA a better assay. Unfortunately, antigens used in MIA are not commercially available and the reading systems (Luminex) are not cost-efficient in studies of large numbers of samples required for seroprevalence estimation.

Serological tests based on E protein are less available. Although poor or null DSp is obtained using a whole DENV-E protein in MIA (Wong et al., 2017), its performance significantly improves using EDIII, suggesting that this epitope is less cross-reactive. Similarity, the DSp of anti-ZIKV EDIII IgG MIA was 90% in sera from patients with previous flavivirus infections (Rockstroh et al., 2017). Although there is no data on serological tests for DENVs, Modis et al. (2005) reported that EDIII 622–637 peptide is a DENV-specific sequence, indicating that it is a potential candidate for serological tests in formats with high optical resolution.

Other serological methods are the indirect Immunoflourescent Test (IIFT) and rapid diagnostic tests (RDT). IIFT is a microscopic method that can visualize viral proteins expressed in infected cells monolayers *via* antigen–antibody binding. IIFT is commercially available with specificity up to 91% (Arai et al., 2020) reported in dengue patients. However, there are not validation studies conducted in populations with other flavivirus co-circulation including ZIKV. In addition, the use of IIFT for population – scale surveys implies increasing throughput, which seems unfeasible in low and middle-income countries. RDT is a screening method that could provide results at the point-of-care. In a systematic review of 10 studies conducted in dengue endemic areas, the specificity of the dengue RDT IgG component ranges from 65 to 100% (Luo et al., 2019). However, most tests were compared to immunoassays, which present a significantly high false-positive rate, preventing a correct validation (Luo et al., 2019). More robust validation studies involving well-characterized samples from vaccinated volunteers and patients with a well-documented history of flavivirus infections are needed.

The plaque reduction neutralization test (PRNT) is currently the gold standard test to determine previous flavivirus infections. PRNT and its derivatives measure nAbs that block infections of susceptible cell monolayers (Russell and Nisalak, 1967; Russell et al., 1967). However, the presence of potent cross-neutralizing antibodies in recurrent infections (Zompi et al., 2012; Priyamvada et al., 2016; Patel et al., 2017; Durham et al., 2019) affects the specificity of these tests. PRNT shows a DSp of 100% in both, dengue and Zika naïve serum samples, but a total cross-neutralization among secondary dengue infection. In the case of ZIKV PRNT_50_, the DSp is 25% with previous dengue infections (Duffy et al., 2009; Swanstrom et al., 2016; Lee et al., 2018). Although higher cut-off points have been used (e.g., PRNT_60_ or PRNT_90_) (Rainwater-Lovett et al., 2012), to reduce cross-reactivity (Roehrig et al., 2008), it has not been evaluated in endemic areas with more than one flavivirus co-circulation. In addition, these tests usually have a laborious and time consuming methodology, expensive facilities and laboratory supplies, and specialized training, limiting its use in national surveillance of dengue and Zika in endemic countries (Thomas et al., 2009; Lee et al., 2018).

Peptide microarrays represent a powerful tool for mapping type-specific Abs against antigens other than NS1 and E proteins. Using a panel of sera from patients with a well-documented history of different flavivirus infections, immobilized overlapping linear peptides can identify potentially relevant epitopes for surveillance (Viedma et al., 2020). A single 20-residue peptide from ZIKV NS2B was identified in a high-density microarray, that in an ELISA test showed a 95.9% specificity and 96% sensitivity in dengue immune patients (Mishra et al., 2018). More studies are needed to determinate its feasibility and performance for diagnostic and surveillance purposes.

## 7. Conclusion

The short divergence time between DENVs and ZIKV is a major determinant of their structural similarity, explaining a high proportion of cross-reactive over type-specific antibodies, leading to the consideration by some that ZIKV is a “fifth DENV serotype” (Harrison, 2016). Despite the enormous B cell repertoire diversity, some structural constraints imposed by germline IGHV segments may contribute to the induction of cross-reactive and polyreactive antibodies, independently of SHM. In addition, the co-circulation of multiple serotypes and viral species in endemic regions cause sequential heterologous infections, which result in the generation of highly cross reactive responses by clonal selection and extensive SHM in GC’s.

There is a considerable knowledge of structural and immunological E and NS1 protein epitopes, because they represent targets for protective (neutralizing) or disease enhancing antibody responses. However, further research is needed to define novel non-conserved linear epitopes in E (i.e., E DIII) and NS1 proteins, as well as other flaviviral antigens that may be irrelevant in terms of protection or pathology, but sufficiently divergent to increase the specificity of serological tests, regardless of the technological platform. Another challenge is the availability of population cohorts in endemic areas with adequate documentation of previous flaviviral exposure, which is required for extensive validation of test performance and eventual use for seroprevalence estimation. Although cross-reactivity impedes accurate dengue and Zika prevalence estimation, it does not modify decision making in terms of public health intervention, because this is focused on vector control, which in this case is the same for both diseases. However, accurate seroprevalence estimation will be required if widespread vaccination against dengue and Zika becomes a goal.

## Author contributions

CG-C performed bibliographic research, drafted the manuscript, made figures, and table. JM-B and CA-A performed bibliographic research and drafted the manuscript. VO-N and MHR drafted the manuscript and provided significant insight. All authors contributed to the article and approved the submitted version.

## Conflict of interest

The authors declare that the research was conducted in the absence of any commercial or financial relationships that could be construed as a potential conflict of interest.

## Publisher’s note

All claims expressed in this article are solely those of the authors and do not necessarily represent those of their affiliated organizations, or those of the publisher, the editors and the reviewers. Any product that may be evaluated in this article, or claim that may be made by its manufacturer, is not guaranteed or endorsed by the publisher.
